# Supplementary material for: Taming contact line instability for pattern formation
Source: Nat Commun. 2016 Aug 10;7:12458. doi: 10.1038/ncomms12458 (PMC4987529; doi:10.1038/ncomms12458)
Supplement: Supplementary Information — Supplementary Figures 1-8, Supplementary Table 1, Supplementary Notes 1-3 and Supplementary References [file ncomms12458-s1.pdf]

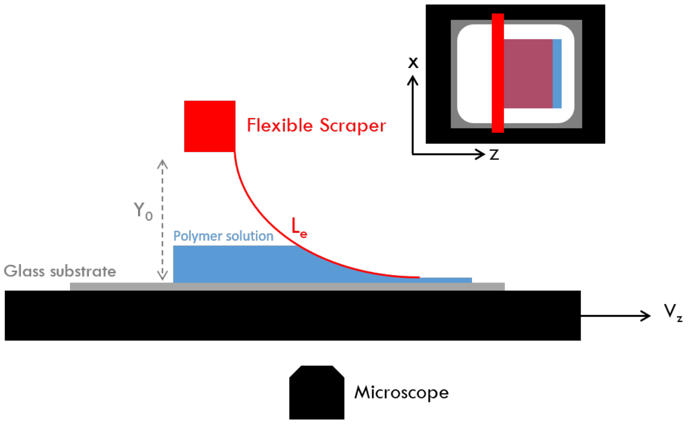

Supplementary Figure 1. Sketch of the experimental setup (not to scale) : it consists of a thin mylar sheet $(0,02 \times 4 \times 3 cm^3)$ held fixed vertically. The spacing y_0 between the glass plate and the upper end of the scraper can be adjusted with a translation stage with $10 \mu m$ precision. The polymer solution is deposited on a hydrophobic glass substrate with dimensions $6 \times 15 cm^2$ and 2 mm thickness. The substrate is then translated in the z direction with a precision motorized translation stage (Marzhauser Wetzlar SCAN IM 130×100) capable of moving at stable speeds ranging from $0.04 mm/s$ to $10 mm/s$. This set up is mounted on an inverted microscope stage.

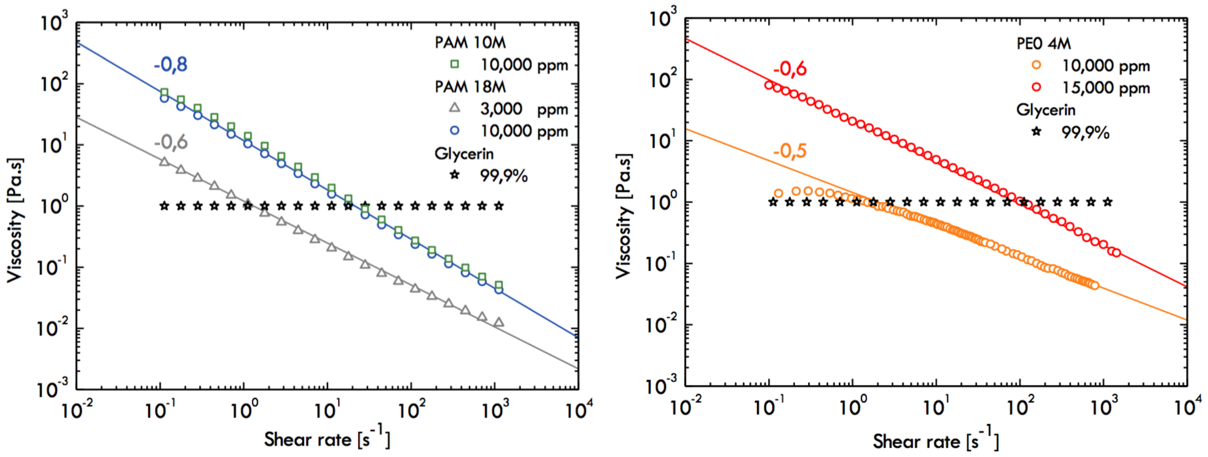

Supplementary Figure 2. Rheology mesurements for polyacrilamide (PAM) and polyethylene oxide (PEO) solutions used at different molecular weights and concentrations.

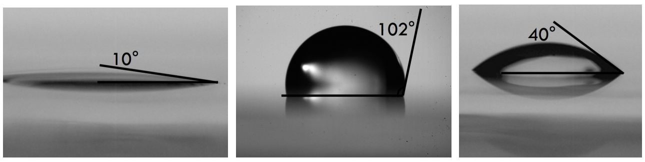

Supplementary Figure 3. Measurement of contact angles on the different glass substrates prepared (from left to right : plasma cleaned, bare and silanized glass) using photographs of a small drop of polymer solution (PAM).

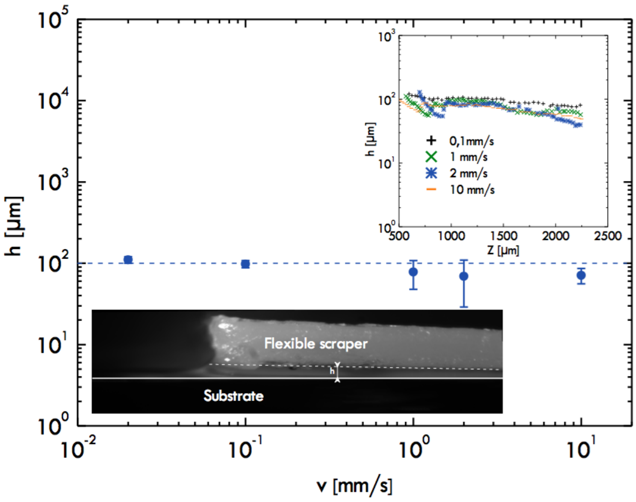

Supplementary Figure 4. Mean distance h between blade and glass surface versus velocity V. This distance is obtained by plotting the position of the bottom side of the blade with respect to the substrate (photograph and inset).

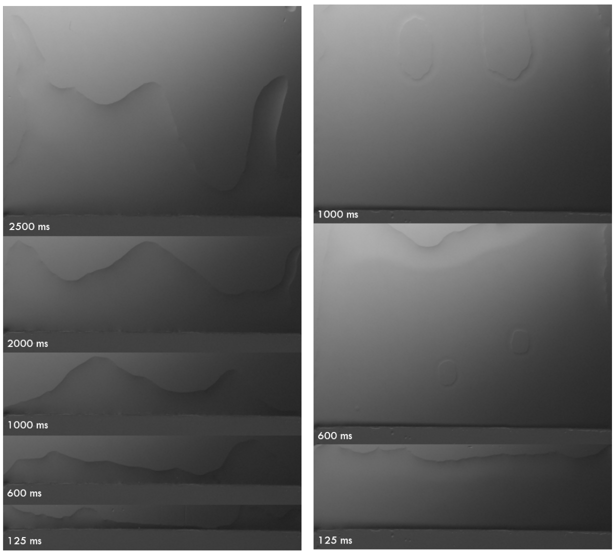

Supplementary Figure 5. Spatio-temporal photographs of the receding contact line for Glycerine (99.9%) for two different translation speeds. Left: Below the transition to film formation $v = 40\mu m/s$. Right: Above the threshold to the transition $v = 100\mu m/s$. Note that dewetting holes can emerge at later times in the deposited film.

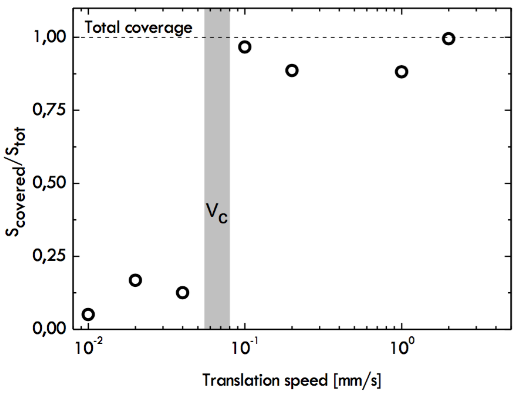

Supplementary Figure 6. Surface covered by Glycerine normalised by the surface explored by the flexible scraper during the coating. The jump from low surface coverage ratio to 1 determines the velocity threshold V_C to film formation (represented by the grey area).

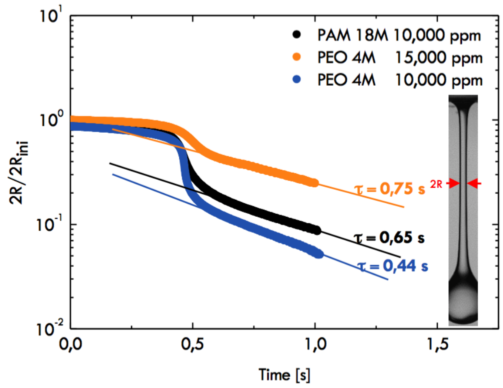

Supplementary Figure 7. Pinch-off experiments of PAM and PEO solutions. The radius of the fluid neck during pinch off is plotted versus time. The later stages can be approximated by an exponential thinning law giving a constant extension rate in the neck. The characteristic times τ of the polymer solutions is obtained from the thinning dynamics and is proportional to the inverse of this stretching rate. The photograph illustrates the detachment of a drop falling from a capillary; note the formation of a long filament to this capillary.

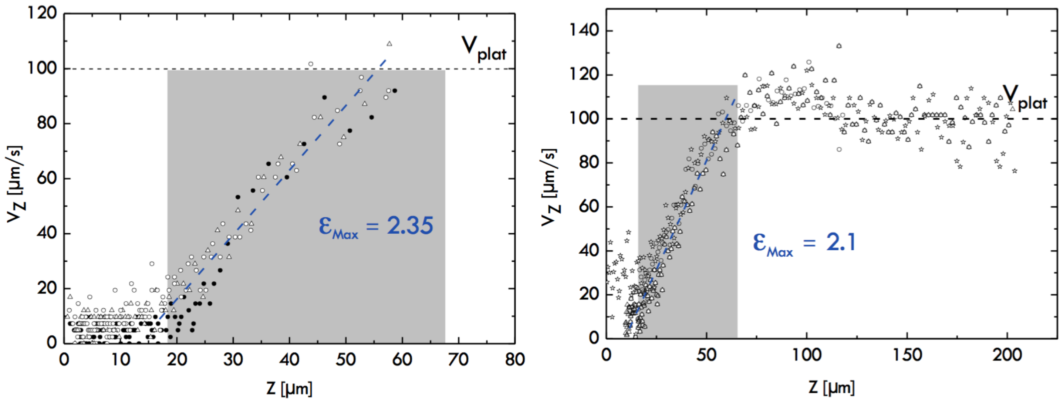

Supplementary Figure 8. Two cases of particle tracking in fingers for PAM (Upper) and PEO (Lower) solutions at the same velocity $v = 100\mu m.s^{-1}$. The stretching rate is obtained from dv_z/dz.

Supplementary table I. Table of contact angles of the different solutions on the surfaces used here.

Solution	Bare Angle (°)	Silanized Angle (°)
**Glycerine**		
99.9%	38	90
**PEO 4M**		
10,000 ppm	20	92
15,000 ppm	20	90
**PAM 10M**		
10,000ppm	20	107
**PAM 18M**		
3,000ppm	22	102
10,000ppm	18	107

Supplementary Notes

Supplementary Note 1. Coating. Experiments carried out using non hydrophobic substrates (plasma cleaned glass or bare glass for which the contact angles are respectively 10° and 45°, the solution forms a homogeneous layer on the substrate for most velocities used. It is known that in dip coating experiments, a transition to a dynamic wetting state giving rise to homogeneous film formation occurs for velocities above a certain threshold. In general, a capillary number Ca=$\eta V/\gamma$ is defined where η is the viscosity of the solution, V is the velocity of the substrate, and γ is the liquid surface tension. The transition to film formation and therefore wetting occurs for capillary numbers which depend on the contact angle, the smaller the contact angle, the smaller the critical capillary number for the wetting transition. Since the viscosity of the solutions used here depends on the shear rate, the capillary number is redefined as Ca=$\eta(\dot{\gamma})V/\gamma$. To estimate the shear rate in these experiments, the distance h between scraper and substrate has to be known. Our visualization experiments during the coating process indicate that this distance is of the order of $100\mu m$ with a small decrease versus velocity (See Supplementary Figure 4). In this case, we neglect shape of the velocity profile and approximate the shear rate $\dot{\gamma} = V/h$. By using this estimate the capillary number varies between 0.01 and 0.1 which is higher than the critical capillary number for the contact angles considered and for which film formation is expected as observed.

In the case where the glass substrate has not been treated with OTS (*i.e*, the contact angles of 45°and 10°) the fingering instability on the receding contact line is therefore not observed in the translation speed range studied, and the coating of the polymer solution always occurs on the glass plate (the value of the critical capillary number that describes the transition for a partially wetting fluid to form a homogeneous film on a surface is near $Ca^* \sim 10^{-2}$. In our case where the viscosity at low shear rates is near $\eta = 50Pa.s$, the transition to film formation should be obtained for a velocity of $v = 0,01mm/s$ (lower than those allowed by our translation stage) indicating that forced wetting is always present. Experiments using Glycerine confirm this conclusion since coating and film formation occurs for speeds as low as $40\mu m/s$ (See the spatio-temporal photograph of the receding contact line of Glycerine at $v = 40\mu m/s$and $100\mu m/s$ in Supplementary Figure 5) corresponding to a critical capillary $Ca_c = 1,56.10^{-3}$, in agreement with the prediction of this value given by the Cox's model [1] :

$$Ca_c = \frac{\theta_e^3}{9ln\frac{L'}{l'}} \tag{1}$$

With L' estimated as the capillary length, l' as the molecular size (nm) and θ_e as the static contact angle (see supplementary table TI)

In order to estimate Ca_c for Glycerine, we used a simple criterion, consisting of the evaluation of the surface covered by the fluid on the substrate $S_{covered}$ during the coating. If the ratio $S_{covered}/S_{total}$ is roughly close to 1, the dewetting speed is lower than the speed of the contact line leading to the total coverage of the substrate (Cf. Supplementary Figure 6). Otherwise, the fluid does not wet the surface and the velocity is below the transition of total coverage. The jump in this ratio $S_{covered}/S_{total}$ allows us to determine the critical velocity V_C of transition from partial wetting to total wetting on the substrate.

However, for equilibrium contact angles greater than 90°, the contact line is unstable and well defined fingers emerge as explained in the main text and as will be described in the next section.

Supplementary Note 2. Determination of the stretching rate in the filaments. The strong dependence of the number of fingers N deposited on the substrate with the velocity is clearly visible in Fig. 1 of the main text. In

Figure 1 (c) of the main text, we reported the mean wavelength of fingers λ (estimated as W/N where W is the width of the blade or the width of the visualization window) deposited on the substrate versus the translation speed. The error bars reflect the variability of the number of fingers (typically on the size of the observation field) for the different videos recorded. We also studied the shape of the fingers as we represented in Figure 2 (main text). In the inset of Figure 2b, we plot the shape of the fingers near the contact line for different spreading velocities (from $50\mu m/s$ to $10mm/s$) and show that the faster the substrate velocity, the wider the fingers. We analyzed the functional shape of these fingers by normalizing the profiles using two characteristic spatial scales (Cf. Fig. 3(b) & 3(c)): a characteristic width l and a characteristic length L. This is shown in Fig.3(b) where the normalized shapes of the fingers do not depend on the translation velocity. Moreover, the finger width l and the finger length L found here increase with the translation speed. We represented in Fig. 3 (c) the dependence of l and L versus the translation speed V, both quantities vary roughly linearly with the translation velocity.
The dependence of the length on the velocity allows to define a characteristic stretching rate V/L. This rate is independent of the velocity V. This property can be rendered more systematic by measuring the mean stretching rate along the fingers by using particle tracking experiments. In order to carry out these measurements, we seeded the solutions with small particles of 1 or 6 μm in diameter (PMMA or Polystyrene fluorescent particles from Polysciences and Molecular Probes) and tracked their trajectories from video imaging. Supplementary Figure 8 below shows the velocity along the center line of the filaments.

In general this velocity is small near the meniscus and then as the particles enter the filament, this velocity increases roughly linearly with distance traveled to reach the translation velocity of the plate. Far away from the meniscus, the filaments are simply translated at the same velocity as the plate on which they are deposited. As mentioned in the main text, the mean stretching rate obtained from dv_z/dz in the centerline of the filaments remains conserved as the velocity changes. Apparently, the filaments all have similar stretching rates for the same solution. This stretching rate changes only when the solution changes. This stretching rate turns out to be determined by the characteristic time of the solutions used. In order to obtain this time scale and as suggested by Hinch, McKinley and others [2], filament stretching experiments or droplet pinch off experiments can be used. Here the thinning rate of the liquid necks connecting the drops to the capillary is controlled by the characteristic time of the solution making this technique useful for its determination. Supplementary Figure 7 below shows the thinning of fluid necks for the different solutions used. Note that the characteristic time scale for the thinning of these necks which follows an exponential law versus time can be obtained by fitting an exponential decay law. From the characteristic time of thinning necks, one obtaines the stretching rate in the neck $\dot{\varepsilon} = \frac{2}{R}\frac{dR}{dt}$ where R is the radius of the liquid neck and t is time. The value of $\dot{\varepsilon}$ is related to the characteristic time of the polymer solutions τ by $\dot{\varepsilon} = 3/\tau$. The value of $\dot{\varepsilon}$ obtained from this measurement is compared to that obtained from the velocity in the centerline of the filaments in the main text (Figure 3 (f)). The two values agree well with each other. The fingers select a constant stretching rate $\dot{\varepsilon}$ fixing the value of the characteristic length L through $\dot{\varepsilon} = V/L$. Because the stretching rate remains constant versus V, the length L increases with V as observed. Since the width of the fingers also increases with L the separation between fingers, and therefore the wavelength of the patterns must increase otherwise the fingers merge by coalescence.

These fingers have a curious dynamical behavior. Two fingers close to each other may merge and form a single finger (see Figure 3 (a) in the main text). This coalescence gives rise to a defect on the linear pattern of lines on the plate. Further and when two fingers drift apart from each other, a third finger may emerge in the spacing between the two initial fingers. We believe this is the mechanism to maintain the spatial wavelength constant: fingers attract and merge and fingers emerge in empty space.

Supplementary Note 3. Details concerning the applications. These patterns have been used for different deposition strategies. Figure 4 shows that if particles (fluorescent from Molecular Probes and not from Polysciences) are embedded into the fluid they may be deposited in a linear pattern with a variable spacing. Further, and if the moving plate is translated in the transverse direction along with its longitudinal translation, wavy and triangular patterns can be created. Larger particles can also be deposited as single isolated spheres or in higher concentrations as seen in the photographs of Fig.4. Another possibility is to use the tunability of the obtained structure both in terms of wavelength and in terms of dimensions to filter different sized particles when they are mixed together in a solution. The supplementary video SV04 illustrates this possibility. This video was recorded in bright field and in fluorescent microscopy and shows an example of the coating of a solution with particles of 1 & $6\mu m$ in diameter. Particles of $1\mu m$ appear bright as they are fluorescent. The small particles are seen to enter the filaments while the larger ones are blocked near the meniscus.

Another observation was made using additional fluorescent DNA molecules added to the solutions. The DNA T4 GT7 (from Nippon Gene) are made fluorescent thanks to the intercalator POPO3 (from Molecular probes). It was added in the proportion of 1 for 5 base pairs. The DNA and polymer solutions are made with TRIS EDTA buffer solution (from Fluka) at pH 8. The total length of fully stretched DNA T4 with fluorescent molecules is estimated as $72\mu m$. Here, the formation of the filaments stretches the DNA molecules which are then deposited on the substrate.

This stretching can be quite important as the length of the molecule shown in Fig. 4 (d and e) is almost 50% of the contour length of the molecule. Stretching deformable objects or aligning anisotropic entities such as carbon nanotubes in these filaments with variable spacing opens the possibility of making anisotropic structures with tunable properties useful for different uses such as screening (biological molecules) or electrical and optical coatings.

Supplementary References

[1] Cox R. G., Journal of Fluid Mechanics, Volume 168, July 1986, pp 169- 194.
[2] Clasen C., Eggers J., Fontelos M. A., Li J. And McKinley G. H. Fluid Mech. (2006), vol. 556, pp. 283â€“308.
